# Integrating views on support for mid-level health worker performance: a concept mapping study with regional health system actors in rural Guatemala

**DOI:** 10.1186/s12939-015-0225-4

**Published:** 2015-10-08

**Authors:** Alison R. Hernández, Anna-Karin Hurtig, Kjerstin Dahlblom, Miguel San Sebastián

**Affiliations:** Division of Epidemiology and Global Health, Umeå University, 901 87 Umeå, Sweden

**Keywords:** Health worker, Nursing, Performance, Relationships, Health services management, Health system, Concept mapping

## Abstract

**Introduction:**

Mid-level health workers are on the front-lines in underserved areas in many LMICs, and their performance is critical for improving the health of vulnerable populations. However, improving performance in low-resource settings is complex and highly dependent on the organizational context of local health systems. This study aims to examine the views of actors from different levels of a regional health system in Guatemala on actions to support the performance of auxiliary nurses, a cadre of mid-level health workers with a prominent role in public sector service delivery. A concept mapping study was carried out to develop an integrated view on organizational support and identify locally relevant strategies for strengthening performance.

**Methods:**

A total of 93 regional and district managers, and primary and secondary care health workers participated in generating ideas on actions needed to support auxiliary nurses’ performance. Ideas were consolidated into 30 action items, which were structured through sorting and rating exercises, involving a total of 135 of managers and health workers. Maps depicting participants’ integrated views on domains of action and dynamics in sub-groups’ interests were generated using a sequence of multivariate statistical analyses, and interpreted by regional managers.

**Results:**

The combined input of health system actors provided a multi-faceted view of actions needed to support performance, which were organized in six domains, including: *Communication and coordination, Tools to orient work, Organizational climate of support, Motivation through recognition, Professional development* and *Skills development*. The nature of relationships across hierarchical levels was identified as a cross-cutting theme. Pattern matching and go-zone maps indicated directions for action based on areas of consensus and difference across sub-groups of actors.

**Conclusions:**

This study indicates that auxiliary nurses’ performance is interconnected with the performance of other health system actors who require support, including managers and community-level collaborators. Organizational climate is critical for making auxiliary nurses feel supported, and greater attention to improving the quality of hierarchical relationships is needed in LMIC settings. The participatory nature of the concept-mapping process enabled health system actors to collaborate in co-production of context-specific knowledge needed to guide efforts to strengthen performance in a vulnerable region.

## Introduction

Mid-level health workers (MLHWs) play a critical role in delivering health care in rural and remote areas [1]. In many low and middle income countries (LMICs), they form the front-line of service delivery for the poorest and most vulnerable populations. MLHWs have a shorter training than professional health workers, though they perform some of the same tasks, and unlike community health workers, they typically have formal certification from nationally-accredited training institutions [2]. In many African and Asian countries, new cadres of MLHWs have been created to fill gaps in underserved areas, such as health extension workers in Ethiopia and lady health workers in Pakistan. While in most Latin American countries, the front-line workforce is made up by a high proportion of auxiliary nurses (ANs), a long established group of MLHWs [3]. In Guatemala, ANs are the largest group of health worker, and they have a prominent role in public sector service delivery in rural areas where professionals are scarce [4].

The performance of MLHWs who are attending vulnerable populations with great health needs is a key leverage point to strengthen health systems’ capacity to redress inequities and enable achievement of the highest attainable standard of health. Realization of these health system goals depends on health worker performance in the dimensions of being available, competent, productive and responsive [5]. Scaling up the numbers of MLHWs has been a key strategy to improve availability in areas with a chronic shortage of professional health workers. However, once in place, they face challenging working conditions characterized by lack of basic resources, sporadic supervision, and delayed wages. Improving health workers’ performance in low-resource settings is a complex endeavor because these intersecting conditions influence both their capacity and motivation to deliver care with competence, productivity and responsiveness.

Due to the interconnections among these elements that shape health worker performance, interventions must be multifaceted and address characteristics of the health worker as well as the health facility and health system [6, 7]. Previous studies in LMIC settings have identified examples of successful implementation of interventions in these areas, such as clinical guidelines, supervision, pay-for-performance incentives, and decentralization of management functions [8–11]. However, the relationships between interventions and the desired changes are complex and highly context-dependent. Meta-analyses indicate that no strategy is effective in all settings, rather their success depends on implementation aspects and organizational processes [12, 13]. A realist review of human resource management intervention studies, which analyzed why certain interventions work in certain contexts and not in others, points to the importance of adaptation to the local situation and involvement of local stakeholders in identifying and implementing solutions to problems [14].

In order to guide the development of context-specific strategies that respond to local conditions and build on local capacities, there is a need for research that gives voice to health system actors’ views of the organizational configurations shaping health service delivery in LMICs [15]. A growing number of studies provide nuanced insight into how health workers and supervisors perceive the influence of challenging conditions and human resource management strategies on performance, motivation and job satisfaction [16–20]. While these studies indicate directions for action, effective design and implementation of interventions to modify conditions and strengthen support for performance depend on the organizational context of the local health system. Additional steps are needed to connect these voices to those of the actors in meso-level management of local health systems who play a critical role in the operational aspects of organizational support. Methodological approaches that integrate the experiential knowledge that groups of local actors draw on when working towards a complex goal, such as improving performance, can help facilitate the translation of health systems research to practice [21].

This study examines the views of actors from different levels of a regional health system in rural Guatemala on actions to support the performance of auxiliary nurses (ANs), in order to identify locally relevant strategies for strengthening performance. Previous studies conducted with ANs and supervisors in this setting indicated constraining factors, such as limited resources, control-oriented supervision and knowledge limitations, as well as factors that enabled their performance, including orientation to their work through community connectedness, and nursing principles of interpersonal relationships and vocation [22, 23]. The aim of this study was to gain insight into the views of health workers, district and regional managers on how these factors could be developed as well as other actions needed to support AN performance.

## Methodology

### Concept mapping

The methodology of concept mapping employs a structured, participatory process and rigorous data analyses to integrate the input of multiple stakeholder groups, and produce maps that depict the composite thinking of organizations or systems [24]. The method was selected based on its capacity to connect diverse actors in co-production of knowledge that is relevant for understanding and potentially transforming the operation of complex human systems [25]. Qualitative and quantitative data are generated and integrated by participants, and then analyzed using multivariate statistical methods to produce visual representations or maps that synthesize the ideas and priorities of groups of actors. The concept mapping process includes the steps of identifying the focus, generating ideas, structuring ideas through sorting and rating, representation in maps, and interpreting the maps. Description of the implementation of these steps follows the orientation to the study setting.

### The study setting

This study was conducted in the department of Alta Verapaz, located in the highlands of northern Guatemala. The population of 1.1 million lives predominantly in rural areas and 90 % belong to indigenous Mayan ethnic groups. Many rural residents are monolingual in the local languages of Q’eqchi’ and Poqomchi’, and have low levels of formal education. Alta Verapaz has the highest level of extreme poverty in the country (38 %), the second highest rate of illiteracy (40 %) [26]. The leading causes of mortality include pneumonia, acute diarrheal diseases, and malnutrition, and the maternal mortality rate is one of the highest in the country [27].

In the national public health system, the department of Alta Verapaz corresponds to a Health Region, which is sub-divided into 19 Municipal Health Districts. The managers working at the Regional Health Office (RHO) are responsible for administration and oversight of the implementation of the Ministry of Health’s programs at the regional level and in the districts. At the district level, health services are provided through two district hospitals, 17 health centers and 34 health posts. Non-governmental organizations contracted by the Ministry of Health also provide a package of essential services via mobile health teams to the most remote populations through the Coverage Extension Program. Service delivery and program implementation are managed at the district level by a district director and a district nurse, as well as technical coordinators who oversee the Coverage Extension Program.

The ANs work in various roles in primary and secondary health care. Secondary care services are provided in health centers, which typically employ around 15 to 30 ANs, depending on the size of the district, as well as the district medical director and district nurse. There are also positions for medical doctors to work in day and night shifts, but in most districts these positions had high turnover and high vacancy. In larger districts, additional professional nurses support the district nurse in oversight of inpatient and outpatient care, and monitoring program implementation. Health centers are also staffed by technicians in the areas of laboratory, pharmacy, and environmental health. Primary health care activities at the community level are delivered through health posts and the Coverage Extension Program. Health posts are staffed by two ANs and an educator. In the Coverage Extension Program, the mobile team of health workers typically consists of a medical doctor or professional nurse, two or more ANs and an educator. In both primary health care settings, work activities depend on coordination with community health volunteers and communication with local leaders.

### Identifying the focus

The Regional Health Office of Alta Verapaz provided the institutional base for the study, and the exact focus was defined in collaboration with the head of the Nursing Unit and the director of the RHO. They identified the disconnect between managers’ and ANs’ views of performance problems in the districts as a key area to explore with the study, because each group felt that the faults of the other were to blame. While managers felt ANs were apathetic and unmotivated, ANs expressed that their work and efforts were unrecognized and unappreciated. Regional Health Office leaders felt that concept mapping could be useful for integrating the perspectives of managers and ANs, as well as other health workers, to develop a more holistic vision of how to improve performance in the districts.

Based in discussions with RHO actors, the researchers developed a study protocol describing the steps of the concept mapping process that included the sampling plan and an informed consent form for participants. Recruitment of participants would be purposive to assure representation of regional and district managers, and health workers, including ANs as well as health professionals and technicians, from a wide range of work settings in the health region. Sampling would also be based on convenience by coordinating participation in the study with existing activities for managers and health workers in order to minimize interruption of their work.

### Generating ideas

Four idea generation sessions were held during scheduled meetings and trainings where managers and health workers would be gathered at the RHO and in the districts. During these sessions, the first author presented results from previous studies carried out in the region, describing local challenges in health service delivery and tendencies found in nursing personnel that enabled them to confront difficult conditions [22, 23, 28]. The purpose and procedure of the concept mapping study were explained, and attendees were asked to write three or more suggested actions in response to the following focus prompt statement: *Name some actions that could be taken or are being taken to support and develop the performance of the nursing staff in the district health services.* The forms were distributed to all present at the sessions. Those who chose not to participate simply did not return the form, though very few chose not to participate. The director of the RHO and the Head Regional Nurse were present in the two sessions held in the RHO, but they were not involved in inviting participation or collecting the forms in order to assure that those present at the sessions did not feel coerced to participate.

**Fig. 1:**
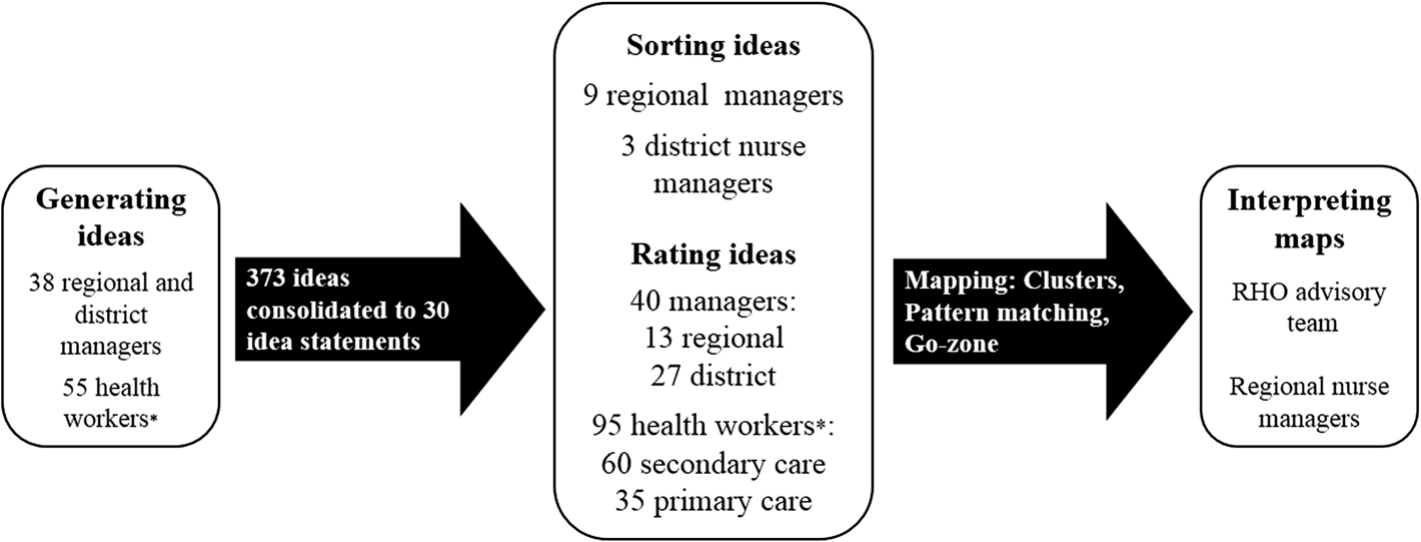
Participation in the concept mapping process. * The majority of health worker participants were ANs, though professional nurses and technicians were also included in this group. RHO = Regional health office

A total of 93 persons contributed ideas, and of these 38 were managers and 55 were health workers (see Fig. 1). All 19 districts were represented among managers and 15 districts were represented among health workers. There were 373 ideas and these were consolidated by the first author by combining repetitions, excluding statements that did not suggest an action, and then organizing the ideas by theme. The thematic consolidated list was discussed with managers from the Nursing and Human Resources Units at the RHO, and 30 action statements were formulated. Agreement on the adequacy of the 30 statements was reached based on the presence of the topics if not the exact content of the original ideas generated, and the need to make the rating survey a manageable length for participants in the following step.

### Structuring ideas: Sorting and rating

Sorting is a technique for determining how participants view interrelationships among the ideas for action from the previous step and identifying organizing themes. The participants were approached on an individual basis in their work setting and were provided with 30 cards with one action written on each card. They were asked to organize the actions into groups that made sense to them and name their groups. The actions that made up the groups were recorded for each participant, and elaborations on the logic of the organization were noted when provided. A total of 12 managers participated in sorting, and two units of the RHO and two districts were represented.

The 30 actions were also used to create a rating sheet, on which health workers and managers were asked to assess the importance of each action based on its potential impact on performance and provide a score from one to five, where five represented great importance and high priority and one represented low importance and low priority. Representation of regional and district managers as well as primary and secondary care health workers was obtained through various means. Regional and district managers completed the ratings during a scheduled meeting at the RHO. Forty managers participated, and all 19 districts and five units of the RHO were represented. District nurses, technical coordinators from the Coverage Extension Program, and regional nurse managers assisted in data collections by administering the rating sheets to health workers in the districts. The sample included 95 health workers from 15 districts, and of these, 60 worked in secondary care and 35 in primary care.

### Representation in maps

The data gathered in the structuring step was analyzed using concept mapping techniques that facilitate visualization of thematic clusters, comparison of patterns in the priorities of sub-groups, and identification of areas of consensus for action [29]. Analysis of the sorting data to generate cluster maps involved three steps: creating a similarity matrix that depicts the number of times pairs of action statements were grouped together, multidimensional scaling to configure points representing the statements on a bivariate plot based on their similarity, and hierarchical cluster analysis to aggregate statements that represent similar concepts into clusters. The most appropriate number of clusters was initially determined through examination of the conceptual coherence of the statements grouped together at successive levels of clustering and evaluation of the relative value of the precision provided by each level of division. Six clusters were identified and names were selected from those suggested by participants in sorting based on their consistency with the content of the clusters.

Rating data was analyzed through comparison of role-stratified averages of the six clusters of actions and the 30 items. R-values were used to evaluate the correlation of sub-groups’ views on the relative importance of action in the different thematic clusters as well as individual items. The greatest divergence in priorities at the cluster level was found in comparing managers and health workers, and these differences are represented in a Pattern Matching map. The greatest divergence in interest in individual action items was found between primary care workers and managers, while the interests of secondary care workers and managers were more highly correlated. These dynamics are represented in Go-zone maps, which depict average importance ratings of individual action items by two different groups on an X-Y bivariate plot, with the respective averages serving as their respective x- and y-coordinates. The X-Y plot is divided into quadrants by lines crossing each axis at the mean importance score of all actions by the respective group, so that items plotted in the top-right quadrant, the Go-zone, represent points of consensus for action.

### Interpreting the maps

The results of these analyses were presented in two forums: a workshop for regional nursing managers, and a meeting of the RHO advisory team, made up by heads of the units. In the workshop, the participants worked in small groups to evaluate the appropriateness of the clusters generated by the analysis and were asked to determine if some actions should be moved to another cluster, if clusters could be joined or divided and if the names were appropriate. Participants reflected on their own experiences with actions to improve performance and discussed connections among the clusters of actions. After the presentation of the maps in the RHO advisory team meeting, members reflected on the accuracy of the depiction of the dynamics across organizational levels, and discussed factors influencing implementation of prioritized actions.

### Ethics, consent and permissions

In accordance with the guidelines of the Guatemalan medical association, ethical clearance was not required as the study did not involve clinical trials or human testing. The study protocol was reviewed and authorized by the head of the Nursing Unit and the director of the RHO, and close contact with regional ministry stakeholders in planning and implementation helped ensure that local norms were respected. Informed consent forms were signed by participants in each step of the study, and they were oriented to the study purpose, measures to protect anonymity, and the voluntary nature of participation.

## Results

### Actions needed to support AN performance

The diverse range of actions suggested for supporting ANs provided a multifaceted view of performance based on the combined input of health workers and managers. The consolidated list of 30 actions (Table 1) included actions aimed at the ANs themselves, as well as managers, and community supporters, which reflected recognition of the interconnection of their performance with other actors in their work environment. Direct actions for supporting ANs included accompanying them and being receptive when they ask for help (item 24), and providing training on sensitivity in human relations as well as standards of practice and ministry programs (items 11, 16). Suggestions were also directed to the work of regional and district managers, such as accompanying them in problem-solving and improving the use of information in decision-making (items 3, 5). Community-focused actions to support ANs’ performance included improved communication with leaders and strengthened training for volunteers (items 8, 14). These suggestions to promote support for managers and communities indicated their perception that better addressing their needs and interests also contributed to ANs’ capacity to perform. The nature of the actions suggested also demonstrates recognition that performance is multidimensional and it depends on nurses’ knowledge of institutional guidelines (item 11), opportunities for professional development (items 17, 19), their ability to relate to patients and communities and speak their language (items 8, 15, 16), as well as their motivation which is influenced by timely payment of wages and formal recognition (items 21, 28, 30).

**Table 1:** Consolidated list of action statements and average rating scores organized by cluster

Clusters		Action items	Average rating
Tools to orient work	1	Provide orientation and induction for new employees	4.32
	2	Promote monitoring of quality of care	4.26
	3	Accompany the districts – do not just point out problems, rather understand and support them	4.11
	4	More supervision of work at the community level including suggestions on how to work better in the communities	4.09
	5	Fortify district managers’ capacity to utilize information to guide decision-making	4.02
	6	Personalized orientation to humanitarian aims of work – not just productivity	3.91
	7	Provide guidelines for implementation of monitoring for employees at the district level	3.80
Communication and coordination	8	Improve communication with community leaders and the community so that we work as a team with better coordination	4.17
	9	Promote team work by delegating responsibilities and authority, and recognizing the importance of the contribution of all	4.15
	10	Accompaniment from district and regional management in some community meetings to promote trust in the services	3.95
Skills development	11	Trainings in the standards of practice and programs of the Ministry	4.45
	12	Continuing education meetings in the districts with themes that respond to detected needs	4.12
	13	Promote the use of technology to facilitate communication and efficient use of information	4.00
	14	Fortify trainings for community team with support from the district and educational materials	3.97
	15	Classes in Q’eqchi/Poqomchi for personnel who are not proficient in the local language	3.97
Professional development	16	Sensitivity trainings for personnel on empathy, trust and respectful treatment to promote good human relations	4.29
	17	Facilitate support to continue studies with permissions from the regional health office	4.11
	18	Strengthen training in vocation in the local nursing school	4.06
	19	Opportunities for development through short courses	4.05
	20	Develop nursing forums where nursing leaders share their vision and accomplishments to promote identification with the profession	3.56
Organizational climate of support	21	Negotiate for the timely payment of monthly wages	4.56
	22	Treat personnel with respect – do not speak to them in a derogatory way and value the psycho-social human being	4.53
	23	Promote climate of trust and mutual support through positive leadership at all levels	4.06
	24	Accompany nursing personnel: be attentive to their needs, resolve their doubts, and be receptive when they ask for help	4.01
	25	Recognize and support the actions carried out at the local level to obtain resources, develop projects and coordinate transport	4.00
Motivation through recognition	26	Recognize positive aspects like dedication, quality of service and connection to the population	4.15
	27	Recognition of actions that contributed to a saved life	4.13
	28	Management should recognize our work through verbal and written congratulations	3.98
	29	Recreational activities with personnel to promote better interpersonal relationships	3.84
	30	Recognize an employee of the month with a certificate	3.62

### Interpreting domains of action

**Fig. 2:**
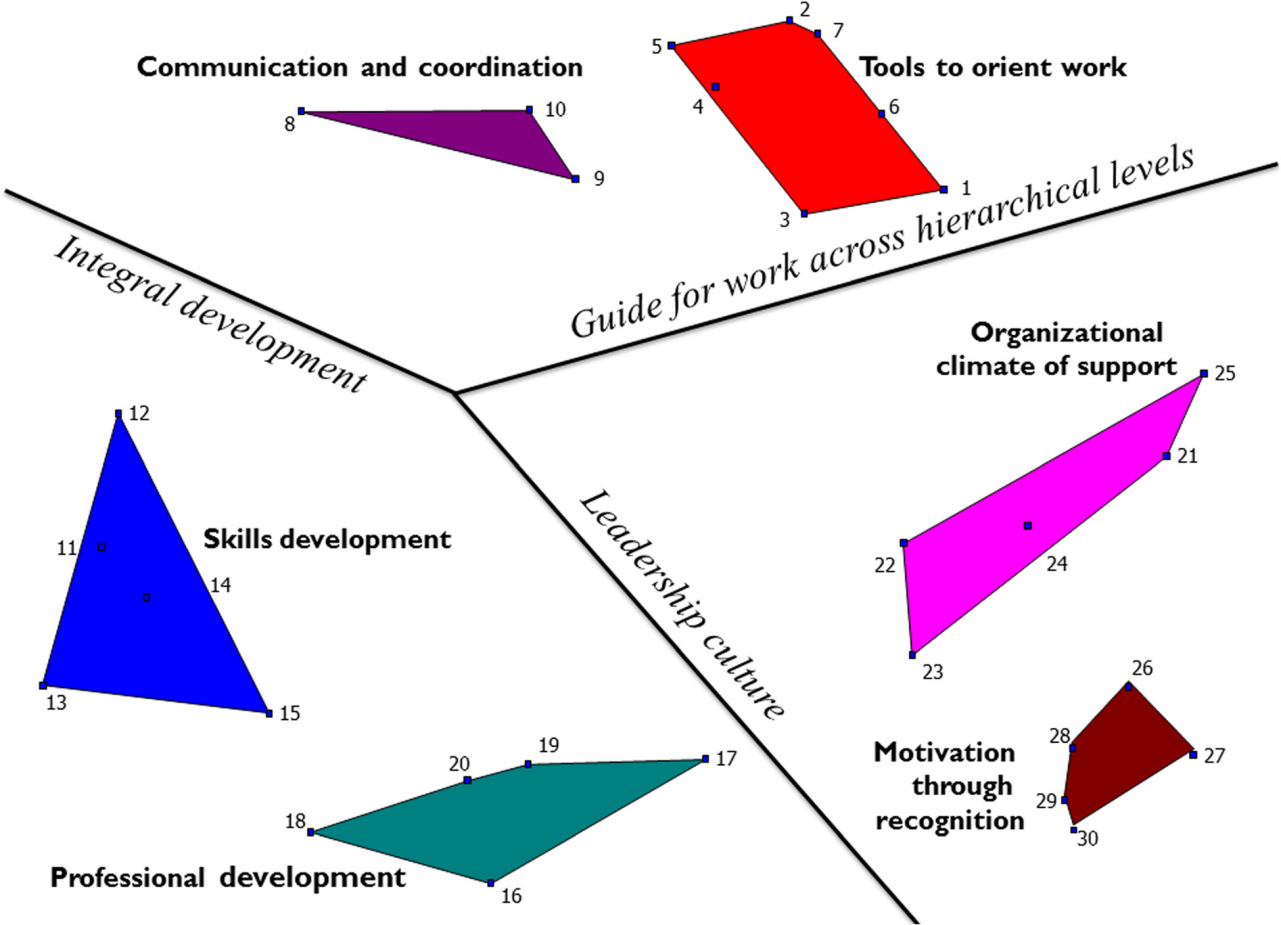
Cluster map divided by regions

Analysis of the sorting data indicated six thematic domains of actions which captured different forms of support that contribute to performance: *Communication and coordination, Tools to orient work, Organizational climate of support, Motivation through recognition, Professional development* and *Skills development*. These clusters and the action items of which they are comprised are depicted in the cluster map in Fig. 2. Nursing managers from the RHO who participated in the workshop to evaluate the clusters confirmed the appropriateness of the cluster names and the comprehensiveness of these thematic domains. The six-cluster organization was used in the comparative analysis of the priorities of managers and health workers presented in the following section of the results.

Feedback from the workshop and field notes from individual sorting sessions also provided a base for further interpretation of the clusters and allowed for identification of three regions of the map (shown in Fig. 2). The clusters *Communication and coordination* and *Tools to orient work* were seen to be related in providing a “Guide for working across hierarchical levels”. The clusters *Professional development* and *Skills development* represented actions for “Integral development”. Participants pointed out that integral development for nurses included technical capacity as well as social capacities, such as sensitivity in human relations with patients and a sense of vocation. The clusters *Organizational climate of support* and *Motivation through recognition* were seen as aspects of “Leadership culture”, which influence the affective state of workers. Items in *Organizational climate of support* could be divided into actions that contribute to the psycho-social environment and actions to resolve problems or address needs. While actions to establish a positive psycho-social environment provide a base for working relationships, actions to resolve problems serve as responsive feedback to the workers, reinforcing that their needs are understood and considered important. Based on this division, actions promoting *Motivation through recognition* were also seen as related to the psycho-social environment of the organization.

Workshop participants also emphasized that the clusters were interrelated. They perceived that the *Organizational climate* of working relationships would shape the way actions in the cluster *Tools for orienting work*, such as induction for new employees and monitoring, were implemented and their impact. The provision of opportunities for *Professional* and *Skills development*, in line with the needs detected through application of the *Tools for orienting work*, would demonstrate responsiveness and contribute to an *Organizational climate of support*.

The nature of relationships across hierarchical levels was identified as a cross-cutting issue that was central to performance. Workshop participants expressed that relationships between patients and ANs, ANs and district managers, and district managers and regional managers operate in a chain reaction. They described that the satisfaction of the patient begins with the ANs’ sense of well-being, which is influenced by their relationship to their managers. Treating personnel with respect, valuing their psycho-social well-being, being attentive to their needs and recognizing their contributions were actions that helped ANs feel good in their work (items 22, 24, 26, 27, 28). In the same way, district managers’ sense of well-being is shaped by their relationships to regional managers through the way they are treated and the nature of the support they receive to perform their role (items 3, 6). The regional nurse managers pointed out that because the nature of relationships operates in a chain reaction, it was possible to improve patients’ satisfaction by modelling respectful treatment and responsive support at the top level of the regional health system.

### Identifying and comparing priorities

Analysis of the importance ratings of these 30 actions indicated that items in the cluster *Organizational climate of support* received the highest average rating (4.23) while actions in *Motivation through recognition* had the lowest average rating (3.94). The average importance ratings of items in the clusters *Tools for orienting work, Communication and coordination*, and *Skills development* were very similar (4.07, 4.09 and 4.10, respectively), while *Professional development* was somewhat lower (4.01). The list of clusters, the actions they contain and their respective ratings are presented in Table 1. At the level of individual items, it is particularly noteworthy that the most highly rated action was to “Negotiate for timely payment of wages,” which indicates that responsiveness to health workers’ needs should begin with efforts to alleviate the unfair burden of working without payment for months at a time. These results reflect the average rating of importance by all participants. However, examination of the stratified ratings revealed that sub-groups of health system actors held different views of what support was most needed.

**Fig. 3:**
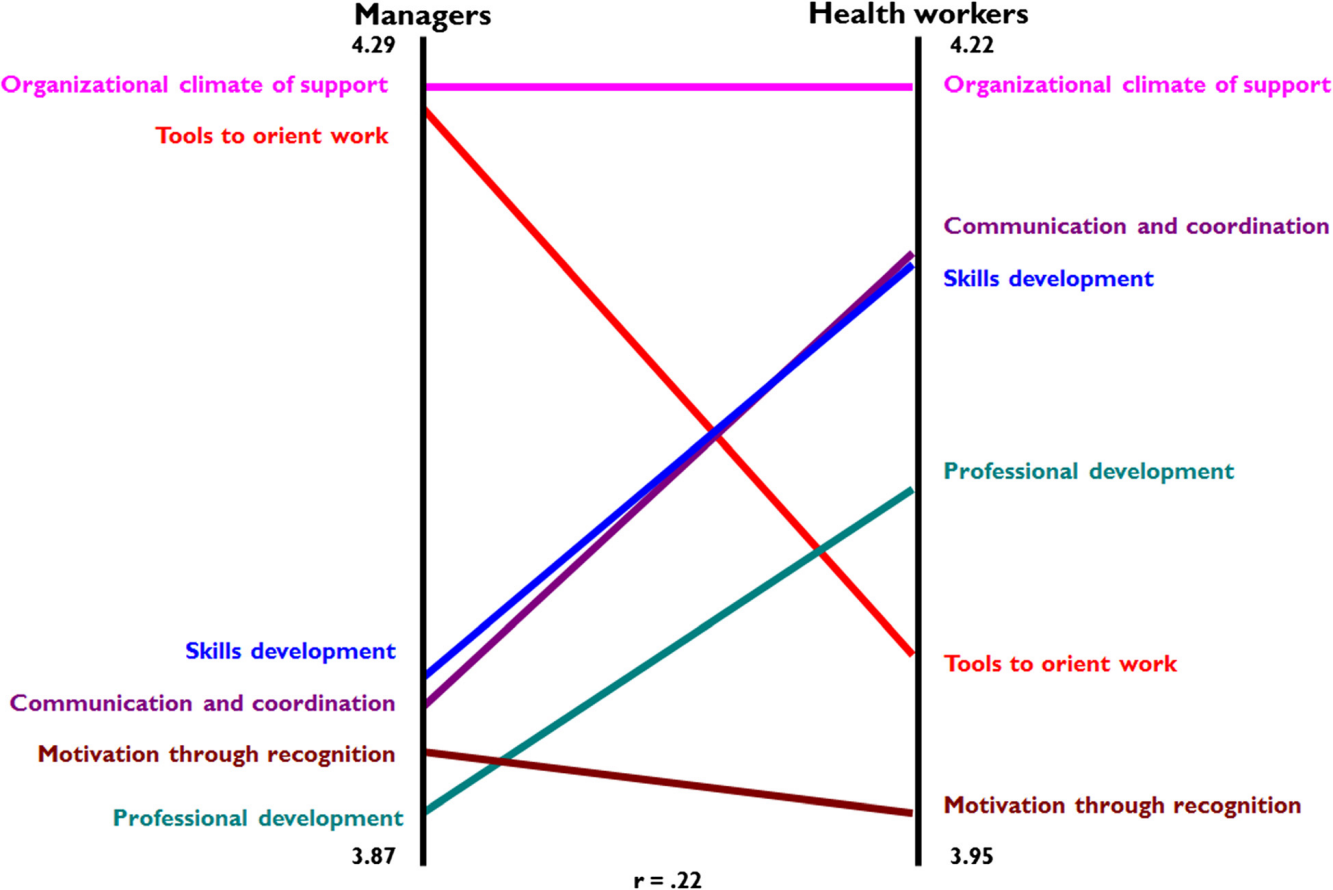
Comparison of prioritization of clusters of actions by managers and health workers

The priorities of managers and health workers are compared at the cluster level in the Pattern Matching map shown in Fig. 3. The cluster *Organizational climate of support* was rated highest and *Motivation through recognition* was among the lowest for both managers and health workers. These patterns indicate that interest in developing a more supportive organizational climate is shared across groups, providing a strong base for prioritization of action in this area. *Tools to orient work* were more highly valued by managers, while *Communication and coordination, Skills development* and *Professional development* were more valued by health workers. Thematic areas of action valued by managers and health workers indicate general directions for efforts to target the interests of these different groups, though understanding of dynamics in interest is enhanced by examining groups’ ratings of the individual items within the clusters.

**Fig. 4:**
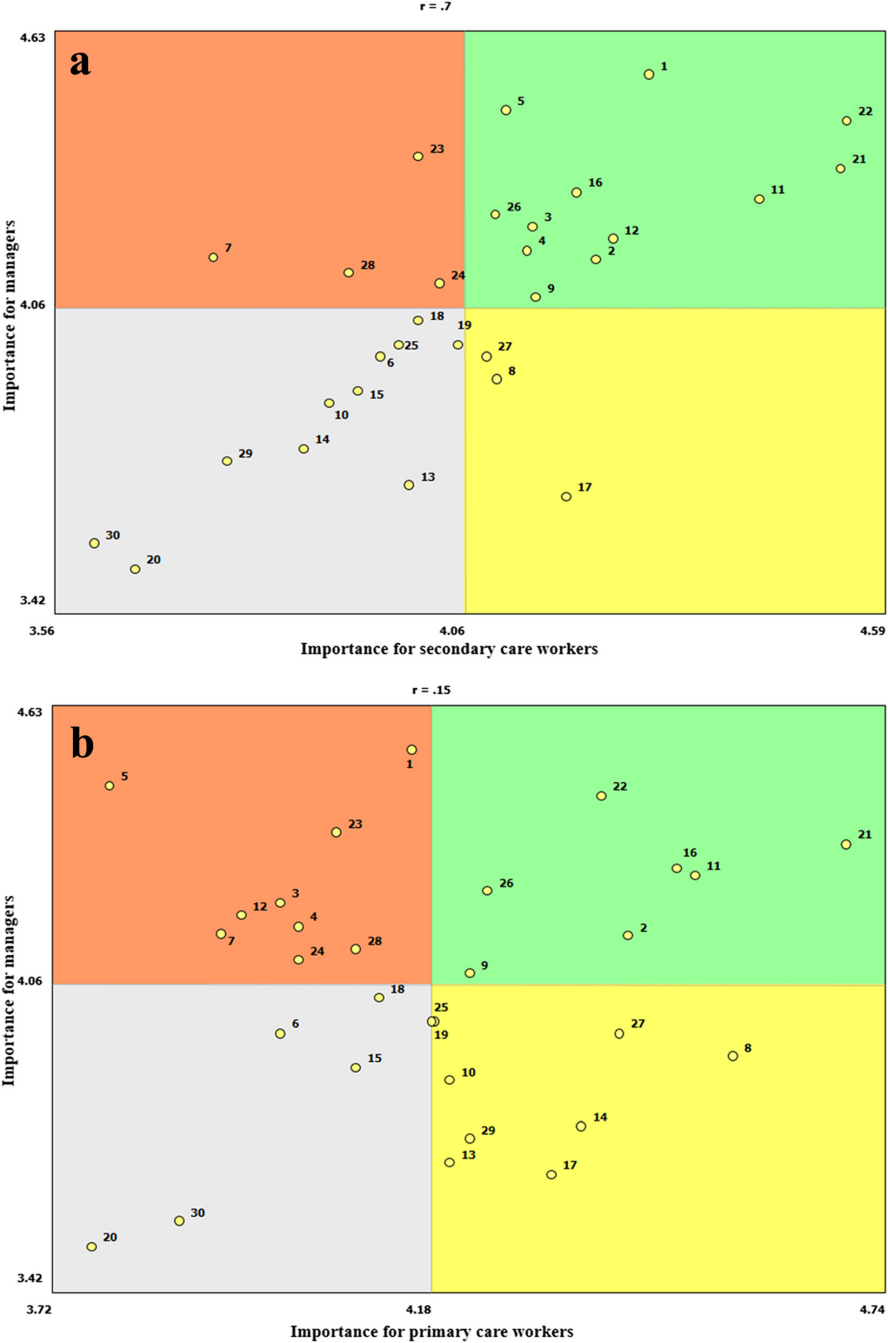
Go-zone maps (**a**). Importance rating of items by secondary care workers and managers (**b**). Importance rating of items by primary care workers and managers

Comparison of the rating of individual actions by the sub-groups of primary and secondary care health workers and managers provided insight into dynamics in the kinds of support most valued in this context. The Go-zone maps (Fig. 4) show actions that were given above average ratings by both the indicated sub-groups of health workers and managers plotted by item number in the upper right quadrant. Comparison of Fig. 4a and b indicates that the majority of actions that were highly valued by secondary care workers were also rated highly by managers. However, many of the actions that primary care workers considered important, particularly for supporting performance at the community level and recognizing the contribution of leaders and volunteers (items 8, 14, 10), were less valued by managers.

## Discussion

This study presented an integrated view of regional health systems actors’ perspectives on actions needed to support the performance of front-line ANs in a rural department of Guatemala. Locally-proposed actions addressed diverse forms of support for ANs including skills development, professional development, managerial tools, improved communication, organizational climate and positive recognition. These domains of action correspond to many of the challenges and barriers to performance identified in studies with health workers and managers in other LMIC settings [16, 17, 30–32]. The ideas generated provided a multidimensional view of performance that encompassed technical knowledge and skills, as well as the quality of the interpersonal relationship with the patient and community, and motivational aspects. Inclusion of actions to support managers and community-level collaborators reflected recognition of the interconnection of ANs’ performance with other actors in their work environment. While the need for development of managers’ capacity to improve health worker performance has been recognized in several studies [17, 33, 34], few have emphasized the importance of organizational support for community-level actors [35].

Regional managers’ interpretation of the domains of action indicated that the nature of relationships across hierarchical levels was a cross-cutting issue for supporting performance. The importance of inter-personal relationships for health worker motivation and satisfaction has been lifted up in previous studies which describe recognition, appreciation, trust and respect as essential attributes [32, 36, 37]. This study provided further description of the nature of relationships that support performance, including attention to psycho-social well-being, understanding of needs and responsive assistance in problem resolution. These qualities were seen as relevant for relationships between managers and nurses, as well as regional and district managers. Regional managers further elaborated on the centrality of relationships to performance, pointing out that the climate of working relationships shaped the way managerial actions were implemented and received, which influenced the AN’s sense of well-being and ultimately patient satisfaction. This interpretation reflects the common sense understanding that how you are treated affects how you treat others, but it also captures how organizational behavior is fundamentally shaped by human interactions and relationships [38, 39].

The centrality of working relationships was also highlighted by the consensus across manager and health worker groups that actions contributing to an “organizational climate of support” were most important for the performance of ANs. The concept of organizational climate has been defined as “the atmosphere that employees perceive is created in their organization by (management) practices” [40]. Health workers’ desire for a more supportive organizational environment has been highlighted in previous studies, and the association between organizational climate and health workers’ satisfaction in their job have been documented previously in LMIC settings [20, 31, 37, 41]. Focus on management of relationships and relationship building has been promoted as an approach to improving the quality of health care work environment, strengthening health workers’ perception of organizational support and their commitment to organizational goals [42]. Nursing research from high income countries has shown that people-focused leadership practices oriented to understanding employees, building trust and responding to employee concerns contribute to better outcomes in job satisfaction, productivity and effectiveness than task-oriented leadership practices [43]. The nature of leadership and management practices that contribute to development of an organizational climate of support in LMIC health system settings is an important focus for further research.

Analysis of interests across sub-groups of health system actors provided a unique view of the social dynamics of this organizational context. Thematic patterns shown in the Pattern-Matching map indicated difference in the kinds of support most valued by health workers and managers. Primary and secondary care health workers were united in their interest in developing professionally, enhancing their skills and improving communication in the workplace, while managers gave more value to tools to orient work. This pattern fits the expectation that the groups would be more interested in support relevant to their own roles. Analysis of sub-groups’ evaluation of individual items in the Go-Zone map revealed divergence in the interests of primary care workers and managers, and greater similarity in the interests of secondary care workers and managers. This trend may be due to the fact that managers have more contact with the work environment and service delivery model of secondary care, while the primary care model is developed in coordination with remote communities and receives limited direct supervision. However, this finding also reflected that strengthening collaboration with the community was outside the scope of managers’ interests and as such, was not a high institutional priority. Managers’ low evaluation of the importance of actions to support community-level work reflect that local institutional priorities are inconsistent with national health policy, which specifies that “organized communities that prioritize actions for health promotion and prevention” are the functional base for comprehensive health care [44]. The tension between policies that prioritize health promotion and prevention and institutional practice has also been identified in other Latin American settings [45].

### Methodological considerations

Recognition of the complexity of health system change has led to increasing interest in systems-oriented methodological approaches that consider dynamic relationships among system components and integrate the experiential knowledge actors draw on when working towards a shared, complex goal [46, 47]. Concept mapping is a recognized system-based tool for gaining understanding of complex issues in human and organizational systems in order to guide planning and evaluation of action for change [24, 25]. However, application of the method in LMIC health system settings is limited [21, 48, 49], and this study illustrates its usefulness for engaging with local actors in a collaborative research process in order to generate context-specific knowledge of a complex phenomenon.

Concept mapping was used to gain a meso-level system view of how to support ANs’ performance, and this study benefited from the method’s unique strengths. The multi-step participatory process allowed for integration of the views of diverse groups through qualitative idea generation and quantitative structuring and evaluation of ideas for action. Local actors’ roles in co-producing the items that formed the content of the study and in interpreting the findings contrasts with other studies where the researchers traditionally take on these roles [50]. Interpretation by regional managers was facilitated by the visualization of conceptual themes and group dynamics in cluster, pattern-matching and go-zone maps. The active involvement of local actors throughout the process and the broad participation of managers and health workers from almost all districts in the region supported the validity of the representation of the regional health system in the findings. While there was a greater proportion of managers represented than health workers, role-stratified analysis ensured that their views on the relative importance of proposed actions were given equal weight in the findings. Further stratification of the findings by district could provide regional managers with a more nuanced view of variation in performance support needs within the health region, however, such analysis was beyond the scope of this study.

There were some challenges in applying this method in a LMIC regional health system setting. In other examples of concept mapping studies in the literature, the steps are often conducted over multi-day workshops and/or via internet platforms. These options were not feasible for this setting. The participation of health workers from geographically disperse districts was accomplished through coordinating idea generation sessions with existing meetings and enlisting managers to assist in administration of ratings surveys with health workers in the districts. It could be possible that health workers felt pressure to participate or hide their real opinions, due to the role of managers in facilitating, even though they were told it was voluntary and anonymous, and they signed informed consent forms. This may have led some to fill in the form with little personal interest, and provide answers that did not reflect their views. However, given the pattern of variation between health worker and manager responses, this did not seem to be the case.

Participation in the interpretation sessions was limited to regional managers due to the time demand of convening district actors involved in service delivery for a special session. Lack of involvement of health workers and district managers in interpretation was the main limitation of this study, as their insights into the organization of the domains and the operation of support across levels were not included in the final results. In spite of these challenges and limitations, a substantial level of participation was achieved in this study due to the RHO leadership’s interest in the topic and the results. Their involvement in the interpretation of the ideas and priorities expressed by district level actors can contribute to the development of locally-relevant management approaches to support AN performance.

## Conclusions

Regional managers expressed that the results of this study captured the “feeling” of the regional health system, and gave them evidence of what they sensed through experience but was not documented. The conceptual domains of action provided managers with a holistic framework for planning actions to support the performance of front-line ANs based on the integrated views of local health system actors. Dynamics in the actions valued by primary and secondary care health workers and managers indicated that development of an organizational climate of support should take top priority and complementary efforts should attend to interests specific to their different roles in the health system.

Interventions to strengthen the performance of health workers serving vulnerable populations can potentially contribute to redressing health inequalities, but their success depends on their interaction with the organizational configurations of the health systems where they are implemented [12, 15]. Greater understanding of the complex social worlds of health systems, particularly the relationships between health workers, managers and communities, is needed to guide efforts to generate change in system behavior and performance outcomes [51, 52]. This study indicated that concept mapping is a useful methodology for engaging health system actors in co-production of the kind of evidence needed to guide action to improve performance based in understanding of organizational context in LMIC settings.
